# Ranking Support Vector Machine with Kernel Approximation

**DOI:** 10.1155/2017/4629534

**Published:** 2017-02-13

**Authors:** Kai Chen, Rongchun Li, Yong Dou, Zhengfa Liang, Qi Lv

**Affiliations:** ^1^National Laboratory for Parallel and Distributed Processing, National University of Defense Technology, Changsha, China; ^2^College of Computer, National University of Defense Technology, Changsha, China

## Abstract

Learning to rank algorithm has become important in recent years due to its successful application in information retrieval, recommender system, and computational biology, and so forth. Ranking support vector machine (RankSVM) is one of the state-of-art ranking models and has been favorably used. Nonlinear RankSVM (RankSVM with nonlinear kernels) can give higher accuracy than linear RankSVM (RankSVM with a linear kernel) for complex nonlinear ranking problem. However, the learning methods for nonlinear RankSVM are still time-consuming because of the calculation of kernel matrix. In this paper, we propose a fast ranking algorithm based on kernel approximation to avoid computing the kernel matrix. We explore two types of kernel approximation methods, namely, the Nyström method and random Fourier features. Primal truncated Newton method is used to optimize the pairwise L2-loss (squared Hinge-loss) objective function of the ranking model after the nonlinear kernel approximation. Experimental results demonstrate that our proposed method gets a much faster training speed than kernel RankSVM and achieves comparable or better performance over state-of-the-art ranking algorithms.

## 1. Introduction

Learning to rank is an important research area in machine learning. It has attracted the interests of many researchers because of its growing application in areas like information retrieval systems [1], recommender systems [2, 3], machine translation, and computational biology [4]. For example, in document retrieval domain, a ranking model is trained based on the training data of some queries. Each query contains a group of corresponding retrieved documents and their relevance levels labeled by humans. When a new query arrives for prediction, the trained model is used to rank the corresponding retrieved documents for the query.

Many types of machine learning algorithms have been proposed for the ranking problem. Among them, RankSVM [5], which is extended from the basic support vector machine (SVM) [6], is one of the commonly used methods. The basic idea of RankSVM is transforming the ranking problem into pairwise classification problem. The early implementation of RanSVM [7] was slow because the explicit pairwise transformation led a large number of the training samples. In order to accelerate the training process, [8] proposed a primal Newton method algorithm to solve the linear RankSVM-struct problem without the need of explicit pairwise transformation. And [9] proposed the RankSVM based on the structured output learning framework.

As with the SVM, kernel trick can be used to generalize the linear ranking problem to nonlinear case for RankSVM [7, 9]. Kernel RankSVM can give higher accuracy than the linear RankSVM for complex nonlinear ranking problem [10]. The nonlinear kernel can map the original features into some high-dimensional space where the nonlinear problem can be ranked linearly. However, the training time of kernel RankSVM dramatically grows as the training data set increases in size. The computational complexity is at least quadratic in the number of training examples because of the calculation of kernel matrix. Kernel approximation is an efficient way to solve the above problem. It can avoid computing kernel matrix by explicitly generating a vector representation of data that approximates the kernel similarity between any two data points.

The approximation methods can be classified into two categories: the Nyström method [11, 12] and random Fourier features [13, 14]. The Nyström method approximates the kernel matrix by a low rank matrix. The random Fourier features method approximates the shift-invariant kernel based on Fourier transformation of nonnegative measure [15]. In this paper, we use the kernel approximation method to solve the problem of lengthy training time of kernel RankSVM.

To the best of our knowledge, this is the first work using the kernel approximation method to solve the learning to rank problem. We use two types of approximation methods, namely, the Nyström method or random Fourier features, to map the features into high-dimensional space. After the approximation mapping, primal truncated Newton method is used to optimize pairwise L2-loss (squared Hinge-loss) function of the RankSVM model. Experimental results demonstrate that our proposed method can achieve high performance and fast training speed than the kernel RankSVM. Compared to state-of-the-art ranking algorithms, our proposed method can also get comparable or better performance. Matlab code for our algorithm is available online (https://github.com/KaenChan/rank-kernel-appr).

## 2. Background and Related Works

In this section, we present the background and related works of learning to rank algorithm and RankSVM.

### 2.1. Learning to Rank Algorithms

Learning to rank algorithms can be classified into three categories: pointwise approach, pairwise approach, and list-wise approach.

- Pointwise: it transforms the ranking problem into regression or classification on single objects. Then existing regression or classification algorithms are directly applied to model the labels of single objects. This approach includes McRank [16] and OC SVM [17].
- Pairwise: it transforms the ranking problem into regression or classification on object pairs. It can model the preferences within the object pairs. This approach includes RankSVM [5] and RankBoost [18].
- List-wise: it takes ranking lists as instances in both learning and prediction and can optimize the list-wise loss function directly. This approach includes ListNet [19], AdaRank [20], BoltzRank [21], and SVM MAP [22].

In this paper, we focus on the pairwise ranking algorithm based on SVM.

### 2.2. Linear RankSVM

Linear RankSVM is a commonly used pairwise ranking algorithm [5]. For the web search problem with *n* queries and a set of documents of each query, features **x**_*i*_ ∈ *ℝ*^*d*^ are extracted from the query-document pair (*q*_*i*_, doc_*i*_) and label *y*_*i*_ ∈ *ℤ* is the relevance level of the doc_*i*_ to the query *q*_*i*_. Thus, the training data is a set of label-query-instance tuples (*y*_*i*_, *q*_*i*_, **x**_*i*_). Let *𝒫* denote the set of preference pairs. If (*i*, *j*) ∈ *𝒫*, doc_*i*_ and doc_*j*_ are in the same query (*q*_*i*_ = *q*_*j*_) and doc_*i*_ is preferred over doc_*j*_ (*y*_*i*_ > *y*_*j*_). The goal of linear RankSVM is to get a ranking function

(1) $$\begin{array}{c} f\left(\;x\;\right)=w^{⊤}x \end{array}$$

such that ∀(*i*, *j*) ∈ *𝒫*, *f*(**x**_*i*_) > *f*(**x**_*j*_) = **w**^*⊤*^**x**_*i*_ > **w**^*⊤*^**x**_*j*_, and **w** ∈ *ℝ*^*d*^.

RankSVM has a good generalization due to the margin-maximization property. According to [27], the margin is defined as the closest distance between two data points when the data points project to the ranking vector **w**:

(2) $$\begin{array}{c} d=\min\frac{w^{⊤}\left(x_{i}-x_{j}\right)}{\left\|w\right\|},\;\forall \left(i,j\right)\in P. \end{array}$$

**Figure 1:**
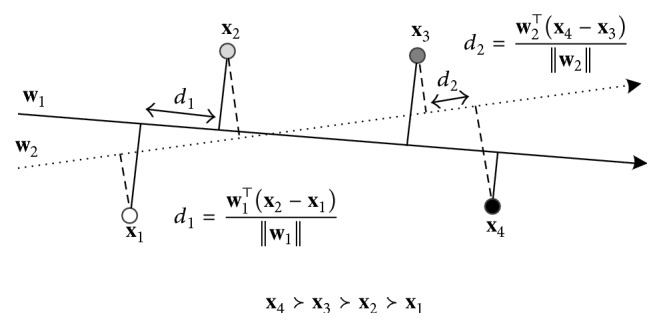
Margin-maximization for linear RankSVM. Four data points have the preference **x**_4_≻**x**_3_≻**x**_2_≻**x**_1_ and can be linearly ranked. *d*_1_ and *d*_2_ are the marginal distances for **w**_1_ and **w**_2_.

Maximizing the margin is good because data point pairs with small margins represent very uncertain ranking decisions. RankSVM can guarantee to find a ranking vector **w** with the maximum margin [27].  Figure 1 shows the margin-maximization of four data points for linear RankSVM. The weights of two linear ranking, namely, **w**_1_ and **w**_2_, can both rank the four data correctly. But **w**_1_ generalizes better than **w**_2_ because the margin *d*_1_ of **w**_1_ is larger than the margin *d*_2_ of **w**_2_.

For L1-loss (Hinge-loss) linear RankSVM [5], the objective loss function is

(3) $$\begin{array}{c} \frac{1}{2}{\left\|\;w\;\right\|}^{2}+C\underset{\left(i,j\right)\in P}{\sum }\max\left(\;0,1-w^{⊤}\left(\;x_{i}-x_{j}\;\right)\;\right), \end{array}$$

where *C* is the regularization parameter. Equation (3) can be solved by standard SVM classification on pairwise difference vectors (**x**_*i*_ − **x**_*j*_). But this method is very slow because of the large size of *𝒫*.

In [8], an efficient algorithm was proposed to solve the L2-loss (squared Hinge-loss) linear RankSVM problem

(4) $$\begin{array}{c} \frac{1}{2}{\left\|\;w\;\right\|}^{2}+C\underset{\left(i,j\right)\in P}{\sum }\max{\left(\;0,1-w^{⊤}\left(\;x_{i}-x_{j}\;\right)\;\right)}^{2}. \end{array}$$

They used a *p* × *n* sparse matrix **A** to obtain the pairwise difference training sample (**x**_*i*_ − **x**_*j*_) implicitly (*p* = |*𝒫*|). If (*i*, *j*) ∈ *𝒫*, there exists a number *k* such that **A**_*ki*_ = 1 and **A**_*jk*_ = −1 and the rest is 0. Let **X** = [**x**_1_,…, **x**_*n*_]^*⊤*^. Equation (4) can be written as

(5) $$\begin{array}{c} \frac{1}{2}{\left\|\;w\;\right\|}^{2}+C{\left(\;1-AXw\;\right)}^{⊤}D\left(\;1-AXw\;\right), \end{array}$$

where **D** is a *p* × *p* diagonal matrix with *D*_(*i*,*j*)(*i*,*j*)_ = 1 if 1 − **w**^*⊤*^(**x**_*i*_ − **x**_*j*_) > 0 and 0 otherwise. Then, (5) is optimized by primal truncated Newton method in *𝒪*(*nd* + *p*).

### 2.3. Kernel RankSVM

The key of kernel method is that if kernel function *κ* is positive definite, there exists a mapping *ϕ* into the reproducing kernel Hilbert spaces (RKHS), such that

(6) $$\begin{array}{c} \kappa \left(\;x,x^{\prime }\;\right)=\langle \;\phi \left(\;x\;\right),\phi \left(\;x^{\prime }\;\right)\;\rangle , \end{array}$$

where 〈·, ·〉 denotes the inner product. The advantage of the kernel method is that the mapping *ϕ* never has to be calculated explicitly.

For L1-loss RankSVM, the objective loss function with the kernel mapping *ϕ* has the form [7]

(7) $$\begin{array}{c} \frac{1}{2}{\left\|\;w\;\right\|}^{2}+C\underset{\left(i,j\right)\in P}{\sum }\max\left(\;0,1-w^{⊤}\left(\;\phi \left(\;x_{i}\;\right)-\phi \left(\;x_{j}\;\right)\;\right)\;\right). \end{array}$$

The primal problem of (7) can be transformed to the dual problem using the Lagrange multipliers.

(8) maxα: ∑ijαij−∑i,j∈P ∑u,v∈PαijαuvQij,uvs.t.: 0≤αij≤C,∀i,j∈P,

where each Langrage multiplier *α*_*ij*_ corresponds to the pair index (*i*, *j*) in *𝒫* and

(9) Qij,uvϕxi−ϕxjϕxu−ϕxv=κxi,xu+κxj,xv−κxi,xv−κxj,xu.

Solving the kernel RankSVM is a large quadratic programming problem. Instead of directly computing the matrix **Q**, we can save the cost by **A** in (5).

(10) $$\begin{array}{c} Q=AKA^{⊤},\;\text{where}\;\;K_{ij}=\kappa \left(x_{i},x_{j}\right). \end{array}$$

The ranking function of the kernel RankSVM has the form

(11) $$\begin{array}{c} f\left(\;x\;\right)=\underset{\left(i,j\right)\in P}{\sum }\alpha_{ij}\left(\;\kappa \left(\;x_{i},x\;\right)-\kappa \left(\;x_{j},x\;\right)\;\right). \end{array}$$

The computation of **Q** requires *𝒪*(*n*^2^) kernel evaluations. It is difficult to scale to large kernel RankSVM by solving (8).

Several works have been proposed to accelerate the training speed of kernel RankSVM, such as 1-slack structural method [9], representer theorem reformulation [27], and pairwise problem reformulation [10]. However, these methods are still slow for large-scale ranking problem because the computational cost is at least quadratic in the number of training examples.

## 3. RankSVM with Kernel Approximation

### 3.1. A Unified Model

The drawback of kernel RankSVM is that it needs to store many kernel values *κ*(**x**_*i*_, **x**_*j*_) during optimization. Moreover, *κ*(**x**_*i*_, **x**) needs to be computed for new data **x** during the prediction, possibly for many vector **x**_*i*_. This problem can be solved by approximating the kernel mapping explicitly:

(12) $$\begin{array}{c} \kappa \left(\;x,x^{\prime }\;\right)\approx \langle \;\tilde{\phi }\left(\;x\;\right),\tilde{\phi }\left(\;x^{\prime }\;\right)\;\rangle , \end{array}$$

where $\tilde{\phi }$ is the mapping of kernel approximation. The original feature **x** can be mapped into the approximated Hilbert space by $\tilde{\phi }$. The objective function of RankSVM with the kernel approximation can be written as

(13) $$\begin{array}{c} \frac{1}{2}{\left\|\;w\;\right\|}^{2}+C\underset{\left(i,j\right)\in P}{\sum }l\left(\;w^{⊤}\tilde{\phi }\left(\;x_{i}\;\right)-w^{⊤}\tilde{\phi }\left(\;x_{j}\;\right)\;\right), \end{array}$$

where *ℓ* is a loss function for SVM, such as *ℓ*(*t*) = max⁡(0,1 − *t*) for L1-loss SVM and *ℓ*(*t*) = max⁡(0,1 − *t*)^2^ for L2-loss SVM. The problems of (13) can be solved using linear RankSVM after the approximation mapping. The kernel never needs to be calculated during the training process. Moreover, the weights **w** can be computed directly without the need of storing any training sample. For new data **x**, the ranking function is

(14) $$\begin{array}{c} f\left(\;x\;\right)=\tilde{\phi }{\left(\;x\;\right)}^{⊤}w. \end{array}$$

Our proposed method mainly includes mapping process and ranking process.

- Mapping process: the kernel approximation is used to map the original data into high dimensional space. We use two kinds of kernel approximation methods, namely, the Nyström method and random Fourier features, which will be discussed in Section 3.2.
- Ranking process: the linear RankSVM is used to train a ranking model. We use the L2-loss RankSVM because of its high accuracy and fast training speed. The optimization procedure will be described in Section 3.3.

The Nyström method is data dependent and the random Fourier features method is data independent [28]. The Nyström method can usually get a better approximation than random Fourier features, whereas the Nyström method is slightly slower than the random Fourier features. Additionally, in the ranking process, we can replace the L2-loss RankSVM with any other linear ranking algorithms, such as ListNet [19] and FRank [23].

### 3.2. Kernel Approximation

#### 3.2.1. Nyström Method

Nyström method gets a low-rank approximation of kernel matrix **K** = [*κ*(**x**_*i*_, **x**_*j*_)]_*n*×*n*_ by uniformly sampling *m* ≪ *n* examples from **X**, denoted by ${\hat{x}}_{1},\ldots ,{\hat{x}}_{m}$. Let $C=[\kappa (x_{i},{\hat{x}}_{j}){]}_{n\times m}$ and $W=[\kappa ({\hat{x}}_{i},{\hat{x}}_{j}){]}_{m\times m}$. The rows and columns of **C** and **K** can be rearranged as

(15) C=WK21,K=WK21⊤K21K22,

where **K**_21_ ∈ *ℝ*^(*n*−*m*)×*m*^ and **K**_22_ ∈ *ℝ*^(*n*−*m*)×(*n*−*m*)^. Then the rank-*k* approximation matrix $\tilde{K}$ of **K** can be calculated as [11]

(16) $$\begin{array}{c} \tilde{K}=CW_{k}^{+}C^{⊤}\approx K, \end{array}$$

where **W**_*k*_^+^ is the pseudo-inverse of **W**_*k*_ and **W**_*k*_ is the best *k*-rank approximation of **W**. The solution of **W**_*k*_ can be obtained by singular value decomposition (SVD) of **W**, **W** = **U**Σ**U**^*⊤*^, where **U** is an orthonormal matrix and Σ = diag⁡(*σ*_1_, *σ*_2_,…, *σ*_*m*_) is the diagonal matrix with *σ*_1_ ≥ *σ*_2_ ≥ ⋯≥*σ*_*m*_ ≥ 0. The solution of **W**_*k*_^+^ can be obtained as

(17) $$\begin{array}{c} W_{k}^{+}=U_{k}\Sigma_{k}^{-1}U_{k}^{⊤}, \end{array}$$

where **U**_*k*_ is the first *k* columns of **U** and Σ_*k*_ = diag⁡(*σ*_1_,…, *σ*_*k*_). Thus, the nonlinear feature mapping of Nyström method can be written as [28]

(18) $$\begin{array}{c} \tilde{\phi }\left(\;x\;\right)=\Sigma_{k}^{-1/2}U_{k}^{⊤}{\left[\;\kappa \left(\;x,{\hat{x}}_{1}\;\right),\ldots ,\kappa \left(\;x,{\hat{x}}_{m}\;\right)\;\right]}^{⊤}. \end{array}$$

The algorithm of the Nyström method is described in Algorithm 1. The total time complexity of the approximation of *n* samples is *𝒪*(*nmk* + *m*^3^). The approximation error of the Nyström method is *𝒪*(*m*^−1/2^) [11].

#### 3.2.2. Random Fourier Features

Random Fourier features is an efficient feature transformation method for kernel matrix approximation by calculating the inner product of relatively low dimensional mappings.

When kernel *κ*(**x**, **y**) is shift-invariant, continuous, and positive-definite, the Fourier transform of the kernel can be written as

(19) $$\begin{array}{c} \kappa \left(\;x,y\;\right)=\int p\left(\;\omega \;\right){exp}^{{j\omega }^{⊤}\left(x-y\right)}d\omega , \end{array}$$

where *p*(*ω*) is a probability density function and *ω* ∈ *ℝ*^*d*^. According to Bochner's theorem [15], the kernel can be approximated as

(20) $$\begin{array}{c} \kappa \left(\;x,y\;\right)=E_{\omega }\left[\;{\tilde{\phi }}_{\omega }{\left(\;x\;\right)}^{⊤}{\tilde{\phi }}_{\omega }\left(\;y\;\right)\;\right], \end{array}$$

where *ω* is sampled from *p*(*ω*). Since *p*(*ω*) and *κ*(**x**, **y**) are real, ${\tilde{\phi }}_{\omega }(x)=\sqrt{2}\cos\left(\omega^{⊤}x+b\right)$ where *b* is drawn uniformly from [0,2*π*] [13]. The expectation in (20) can be approximated by the mean over *m* Fourier components as

(21) $$\begin{array}{c} \tilde{\phi }\left(\;x\;\right)=\sqrt{\frac{2}{m}}{\left[\;\cos\left(\;\omega_{1}^{⊤}x+b_{1}\;\right),\ldots ,\cos\left(\;\omega_{m}^{⊤}x+b_{m}\;\right)\;\right]}^{⊤}, \end{array}$$

where *ω*_*i*_ ∈ *ℝ*^*d*^ is sampled from the distribution *p*(*ω*) and *b*_*i*_ ∈ *ℝ* is uniformly sampled from [0,2*π*]. The algorithm is described in Algorithm 2. The total time complexity of the approximation of *n* samples is *𝒪*(*nmd*). The approximation error of the Nyström method is *𝒪*(*n*^−1/2^ + *m*^−1/2^) [14].

### 3.3. Ranking Optimization

In this section, we solve the L2-loss (squared Hinge-loss) ranking problem of (13) after the kernel approximation mapping of training data

(22) $$\begin{array}{c} \frac{1}{2}{\left\|\;w\;\right\|}^{2}+C\underset{\left(i,j\right)\in P}{\sum }\max{\left(\;0,1-w^{⊤}\left(\;\tilde{\phi }\left(\;x_{i}\;\right)-\tilde{\phi }\left(\;x_{j}\;\right)\;\right)\;\right)}^{2}. \end{array}$$

Similar as (5), the loss function can be rewritten as

(23) $$\begin{array}{c} \frac{1}{2}{\left\|\;w\;\right\|}^{2}+C{\left(\;1-A\tilde{\Phi }w\;\right)}^{⊤}D\left(\;1-A\tilde{\Phi }w\;\right), \end{array}$$

where $\tilde{\Phi }=[\tilde{\phi }(x_{1}),\ldots ,\tilde{\phi }(x_{n}){]}^{⊤}$. The gradient and the generalized Hessian matrices of (23) are

(24) g=w+2CΦ~⊤A⊤DAΦ~w−1,H=I+2CΦ~⊤A⊤DAΦ~,

where **I** is the identity matrix. The Hessian matrix does not need to be computed explicitly using truncated Newton method [8]. The Newton step **H**^−1^**g** can be approximately computed using linear conjugate gradient (CG). The main computation of linear CG method is the Hessian-vector multiplication **Hs** for some vector **s**

(25) $$\begin{array}{c} Hs=s+2C{\tilde{\Phi }}^{⊤}A^{⊤}DA\tilde{\Phi }s. \end{array}$$

Assuming that the embedding space $\tilde{\phi }$ has *m* dimensions, the total complexity of this method is *𝒪*(*nm* + *p*) where *p* = |*𝒫*|. The main step of our proposed algorithm is described in Algorithm 3. We calculate the approximation embedding $\tilde{\phi }$ using the Nyström method or random Fourier features in line (1). Then $\tilde{\phi }$ is applied to all training samples in line (2). The linear RankSVM model with primal truncated Newton method is applied in the embedding space in line (3)–(11).

## 4. Experiments

### 4.1. Experimental Settings

We use three data sets from LETOR (http://research.microsoft.com/en-us/um/beijing/projects/letor), namely, OHSUMED, MQ2007, and MQ2008, to validate our proposed ranking algorithm. The examples of the data sets are extracted from the information retrieval data collections. These data sets are often used for evaluating new learning to rank algorithms.  Table 1 lists the properties of the data sets. Mean average precision (MAP) [29] and normalized discounted cumulative gain (NDCG) [30] are chosen as the evaluation metrics on the performance of the ranking models.

We compare our proposed method with linear and kernel RankSVM as follows:

- *RankSVM-Primal* [8]: it is discussed in Section 2.1 by solving the primal problem of linear L2-loss RankSVM (http://olivier.chapelle.cc/primal/).
- *RankSVM-Struct* [9]: it solves an equivalent 1-slack structural SVM problem with linear kernel (http://www.cs.cornell.edu/People/tj/svm_light/svm_rank.html).
- *RankSVM-TRON* [10]: it solves the linear or kernel ranking SVM problem by trust region Newton method (https://www.csie.ntu.edu.tw/~cjlin/libsvmtools/).
- *RankNystöm*: our proposed RankSVM with the Nyström kernel approximation.
- *RankRandomFourier*: our proposed RankSVM with the random Fourier features kernel approximation.

The hyperparameters of the algorithms are selected by grid search. The regularization parameter *C* of each algorithm is chosen from [2^−12^, 2^−11^,…, 2^6^]. For kernel RankSVM and our approximation methods, the parameter *γ* of RBF kernel is chosen from [2^−12^, 2^−11^,…, 2^2^]. For MQ2007 dataset, the number of sampling for kernel approximation *m* is set to 2000, whereas *m* = 500 for the other datasets. All experiments are conducted on a high performance server with 2.0 GHz 16-cores CPU and 64 GB of memory.

### 4.2. Comparison of the Nyström Method and Random Fourier Features

**Figure 2:**
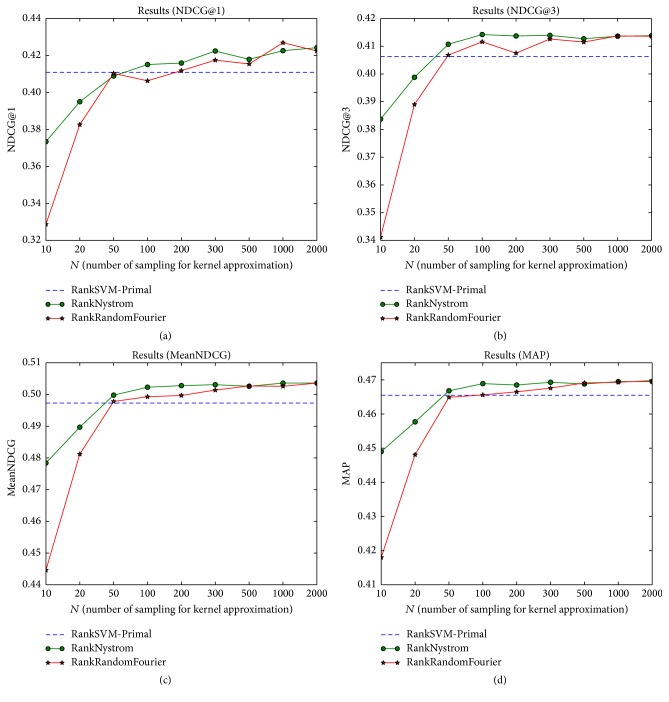
Performance comparison of RankSVM with the Nyström method and random Fourier features on MQ2007 dataset. (a) NDCG@1; (b) NDCG@3; (c) MeanNDCG; (d) MAP.

Figure 2 shows the performance comparison of RankSVM with the Nyström method and random Fourier features on MQ2007 dataset. We take the linear RankSVM algorithm, RankSVM-Primal, as the baseline method, which is plotted as dotted line. The remaining two lines represent RankNyström and RankRandomFourier, respectively. In the beginning, the performances of kernel approximate methods are worse than linear RankSVM. But along with the increase of *m* (the number of sampling of approximation), both of the kernel approximate methods can outperform the linear RankSVM. We also observe that RankNyström gets better results than RankRandomFourier when *m* is small and the two methods obtain similar results when *m* = 2000.

### 4.3. Comparison with Linear and Kernel RankSVM

In this part, we compare our proposed kernel approximation ranking algorithms to other linear and kernel RankSVM algorithms. We take *N* = 2000 for the kernel approximation.  Table 2 gives the results of different RankSVM algorithms on the first fold of MQ2007 dataset. The linear RankSVM algorithms use less training time, but their MeanNDCG values are lower than the values of the kernel RankSVM algorithms. Our kernel approximation methods obtain better performance than the kernel RankSVM-TRON with much faster training speed in this dataset. The training time of our kernel approximation methods is about ten seconds, whereas the training time of the kernel RankSVM-TRON is more than 13 hours. The result of random Fourier features is slightly better than the RankNyström method. Moreover, the L2-loss RankSVM can get better performance than the L1-loss RankSVM on this dataset. The MeanNDCG of RankSVM-Primal (linear) is slightly higher than RankSVM-TRON (linear). The kernel approximation methods get better MeanNDCG than RankSVM-TRON with RBF kernel.

### 4.4. Comparison with State-of-the-Art

In this part, we compare our proposed algorithm with the state-of-the-art ranking algorithms. Most of the results of the comparison algorithms come from the baselines of LETOR. The remaining results come from the papers of the algorithms. The hyperparameters *C* and *γ* of our proposed kernel approximation RankSVM are selected by grid search as in Section 4.1.

Table 3 provides the comparison of testing NDCG and MAP results of different ranking algorithms on the TD2004 dataset. The number of sampling for kernel approximation *m* is set to 500. We can observe that the kernel approximation ranking methods can achieve the best performances on 3 terms of all the 6 metrics. Also, the results of RankNyström and RankRandomFourier are similar.

Table 4 provides the performance comparison on the OHSUMED dataset. *m* is set to 500. We once observe that RankRandomFourier achieves the best performances on 3 metrics of all the 6 metrics. RankNyström gets the best results on 2 metrics.

Table 5 provides the comparison of results on the MQ2007 dataset. *m* is set to 2000. We observe that RankNyström obtains the best scores on 3 metrics on MQ2007 dataset. BL-MART also achieves the best scores on 3 metrics. However, BL-MART trains 10,000 LambdaMART and creates bagged model by randomly selecting a subset of these models, whereas our proposed RankNyström algorithm only trains one model.

## 5. Conclusions

In this paper, we propose a fast RankSVM algorithm with kernel approximation to solve the problem of lengthy training time of kernel RankSVM. First, we proposed a unified model for kernel approximation RankSVM. Approximation method is used to avoid computing kernel matrix by explicitly approximating the kernel similarity between any two data points. Then, two types of methods, namely, the Nyströem method and random Fourier features, are explored to approximate the kernel matrix. Also, the primal truncated Newton method is used to optimize the L2-loss (squared Hinge-loss) objective function of the ranking model. Experimental results indicate that our proposed method requires much less computational cost than kernel RankSVM and achieves comparable or better performance over state-of-the-art ranking algorithms. In the future, we plan to use more efficient kernel approximation and ranking models for large-scale ranking problems.

## Figures and Tables

**Algorithm 1:**
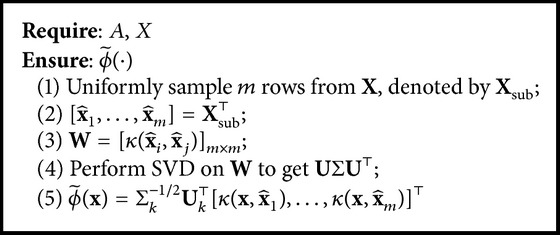
Nyström method.

**Algorithm 2:**
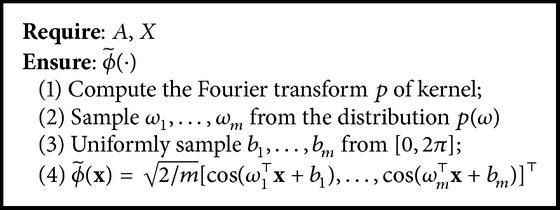
Random Fourier features.

**Algorithm 3:**
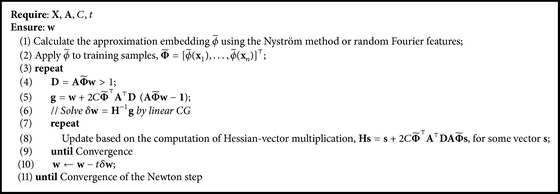
RankSVM with kernel approximation.

**Table 1:** Information of used LETOR data sets. Q-D Pairs denote Query-Document Pairs. Each pair of the data sets has relevance labels in 0 (nonrelevant), 1 (possibly relevant), and 2 (relevant).

Data set	Queries	Q-D Pairs	Features	Relevance
TD2004	75	74,146	64	{0,1}
OHSUMED	106	16,140	45	{0,1, 2}
MQ2007	1692	69,623	46	{0,1, 2}

**Table 2:** Results of different RankSVM algorithms on the first fold of MQ2007 dataset. We take *m* = 2000 for the kernel approximation method.

Algorithm	Type	Loss	*C*	*g*	Mean-NDCG	Time (s)
RankSVM-TRON	linear	L1	2^−5^	—	0.5265	1.9
RankSVM-Struct	linear	L1	2^−1^	—	0.5268	2.2
RankSVM-Primal	linear	L2	2^−10^	—	0.5270	1.2
RankSVM-TRON	RBF	L1	2^−2^	2^−5^	0.5310	47463.5

RankNystöm	RBF	L2	2^−2^	2^−5^	0.5330	10.9
RankRandomFourier	RBF	L2	2^−2^	2^−5^	**0.5336**	16.1

**Table 3:** Performance comparison on TD2004 data set.

	NDCG@1	NDCG@3	NDCG@5	P@1	P@3	MAP
AdaRank-MAP [20]	0.4133	0.4017	0.3932	0.4133	0.3422	0.3308
AdaRank-NDCG [20]	0.3600	0.3838	0.3769	0.3600	0.3289	0.2986
FRank [23]	0.4400	**0.4479**	0.4362	0.4400	0.3867	0.3809
ListNet [19]	0.4400	0.4371	0.4209	0.4400	0.4000	0.3721
RankBoost [18]	0.4800	0.4640	0.4368	0.4800	**0.4044**	0.3835
RankSVM-Struct [9]	0.4400	0.4092	0.3935	0.4400	0.3511	0.3505
RankSVM-Primal [8]	0.4666	0.4468	0.4277	0.4666	0.4000	0.3793

RankNystöm	**0.4933**	0.4348	0.4254	**0.4933**	0.3911	0.3899
RankRandomFourier	**0.4933**	0.4422	**0.4265**	**0.4933**	0.4000	**0.3924**

**Table 4:** Performance comparison on OHSUMED data set.

	NDCG@1	NDCG@3	NDCG@5	P@1	P@3	MAP
RankSVM-Struct [9]	0.5515	0.4850	0.4729	0.6338	0.5898	0.4478
ListNet [19]	0.5326	0.4732	0.4432	0.6524	0.6016	0.4457
AdaRank-MAP [20]	0.5388	0.4682	0.4613	0.6338	0.5895	0.4487
AdaRank-NDCG [20]	0.5330	0.4790	0.4673	0.6719	0.5984	**0.4498**
RankBoost [18]	0.4632	0.4555	0.4494	0.5576	0.5609	0.4411
RankRLS [24]	0.5490	0.4770	0.4530	0.6440	0.5860	0.4470
RankSVM-Primal [8]	0.5645	0.5004	0.4782	0.6710	**0.6112**	0.4439

RankNystöm	**0.5730**	0.4874	0.4780	**0.6801**	0.5890	0.4473
RankRandomFourier	0.5728	**0.4965**	**0.4804**	**0.6801**	0.5983	0.4472

**Table 5:** Performance comparison on MQ2007 data set.

	NDCG@1	NDCG@3	MeanNDCG	P@1	P@3	MAP
RankSVM-Struct [9]	0.4096	0.4063	0.4966	0.4746	0.4315	0.4645
ListNet [19]	0.4002	0.4091	0.4988	0.4640	0.4334	0.4652
AdaRank-MAP [20]	0.3821	0.3984	0.4891	0.4392	0.4230	0.4577
AdaRank-NDCG [20]	0.3876	0.4044	0.4914	0.4475	0.4305	0.4602
RankBoost [18]	0.4134	0.4072	0.5003	0.4823	0.4348	0.4662
LambdaMART [25]	0.4147	0.4119	0.5011	—	—	0.4660
BL-MART [25]	0.4200	**0.4224**	**0.5093**	—	—	**0.4730**
CRR [26]	—	—	0.5000	—	—	0.4660
RankSVM-Primal [8]	0.4109	0.4063	0.4973	0.4747	0.4317	0.4655

RankNystöm	**0.4242**	0.4138	0.5036	**0.4888**	**0.4394**	0.4695
RankRandomFourier	0.4224	0.4136	0.5036	0.4871	0.4386	0.4698
